# Superresolution Imaging of Clinical Formalin Fixed Paraffin Embedded Breast Cancer with Single Molecule Localization Microscopy

**DOI:** 10.1038/srep40766

**Published:** 2017-01-18

**Authors:** Matthew K. Creech, Jing Wang, Xiaolin Nan, Summer L. Gibbs

**Affiliations:** 1 Biomedical Engineering Department, Oregon Health & Science University, Portland, OR 97201, USA; 2 Knight Cancer Institute, Oregon Health & Science University, Portland, OR 97201, USA; 3 OHSU Center for Spatial Systems Biomedicine, Oregon Health & Science University, Portland, OR 97201, USA.

## Abstract

Millions of archived formalin-fixed, paraffin-embedded (FFPE) specimens contain valuable molecular insight into healthy and diseased states persevered in their native ultrastructure. To diagnose and treat diseases in tissue on the nanoscopic scale, pathology traditionally employs electron microscopy (EM), but this platform has significant limitations including cost and painstaking sample preparation. The invention of single molecule localization microscopy (SMLM) optically overcame the diffraction limit of light to resolve fluorescently labeled molecules on the nanoscale, leading to many exciting biological discoveries. However, applications of SMLM in preserved tissues has been limited. Through adaptation of the immunofluorescence workflow on FFPE sections milled at histological thickness, cellular architecture can now be visualized on the nanoscale using SMLM including individual mitochondria, undulations in the nuclear lamina, and the HER2 receptor on membrane protrusions in human breast cancer specimens. Using astigmatism imaging, these structures can also be resolved in three dimensions to a depth of ~800 nm. These results demonstrate the utility of SMLM in efficiently uncovering ultrastructural information of archived clinical samples, which may offer molecular insights into the physiopathology of tissues to assist in disease diagnosis and treatment using conventional sample preparation methods.

Formalin-fixed, paraffin-embedded (FFPE) tissue specimens are the mainstay of tissue archiving with well over 400 million FFPE tissue samples assembled in biorepositories^1^. These preserved tissues have the potential to improve our understanding of diseases, especially in oncology where biopsies and resected specimens are routinely maintained in clinical practice. Use of fresh frozen tissue has long been considered the “gold-standard” for molecular assays as proteins and genetic material are preserved in their native state^2^,^3^,^4^. However, ice crystal formation is common, creating artifacts that are not seen in FFPE preserved tissues^5^. Advances in antigen retrieval techniques have made the majority of proteins accessible in FFPE preserved samples, further establishing it as the standard preservation method for simultaneous analysis by immunohistochemistry (IHC) or immunofluorescence (IF) and histomorphology^6^. Additionally, FFPE preserved tissues are also convenient and cost-effective to archive as they can be stored at ambient temperature with perfectly preserved proteomics for decades^7^.

Histopathological analysis of tissues has a rich history beginning as early as 1858 with the discovery of carmine dyes for staining nuclei in brain tissue sections^8^. While molecular profiling using *in situ* hybridization, IHC or IF, and other techniques are commonly performed on FFPE sections, visualization of morphological features are still most frequently used for disease diagnosis and pathological analysis. However, both morphological and molecular tissue analyses are limited by the spatial resolution of the instrumentation utilized to assess tissue structure as well as genomic, proteomic and metabolomic profiles. Even the best conventional fluorescence microscopes cannot resolve two objects separated by a lateral distance less than half the wavelength of light or, at best, a spatial resolution of ~200 nm^9^. This level of spatial resolution is inadequate to assess intracellular features, masking ultrastructural information that may play an important role in oncological processes such as malignant transformation and therapeutic efficacy. Thus although vast biorepositories of specimens exist, current technology is not capable of spatially assessing functional proteomic and genomic information *in situ* at the molecular, or nanometer, scales.

With the advent of superresolution microscopy (SRM), including techniques based on single molecule localization microscopy (SMLM), visualization of biology at spatial resolutions on the order of 10–20 nm has revealed exciting, new biological phenomena previously invisible to diffraction-limited light microscopy^10^,^11^,^12^,^13^. SMLM has been implemented in two (2D) and three dimensions (3D)^14^,^15^,^16^ and has also been extended to tissue samples, where frozen brain sections were examined using stochastic optical reconstruction microscopy (STORM)^17^, a common form of SMLM. Additionally, single molecules have been imaged in FFPE brain tissue sections overlaid on confocal images providing tissue specific context for single antigens^18^. More recently, stimulated emission depletion microscopy (STED) was used to image FFPE human rectal cancer tissues, the first demonstration of any SRM technique successfully applied to FFPE tissues; the images demonstrated preserved antigenicity for up to 17 years for tissues stored in FFPE blocks^19^. However, to date 2D high-resolution reconstructions of FFPE tissues with SMLM have not been demonstrated and 3D SRM reconstructions of any variety in FFPE tissues have not been shown due to a number of technical hurdles hampering adequate sample preparation and high quality data collection. Thus, SMLM is an additional SRM technique for FFPE imaging that can offer nanoscale resolution in all three dimensions.

Traditionally, high resolution imaging of tissue ultrastructures at the nanometer scale has mostly used serial section transmission electron microscopy (ssTEM), electron tomography (ET-TEM) and serial section scanning electron microscopy (ssSEM)^20^. Recent EM imaging advances that improved tissue ultrastructure assessment include ssSEM with serial blockface scanning electron microscopy (SBF-SEM) and focused ion beam scanning election microscopy (FIB-SEM), which enable automated serial sectioning, improving alignment and reducing overall imaging times^21^. However, EM tissue imaging methods are practically constrained by factors including intricate sample embedding, contrast generation with heavy metal stains such as osmium tetroxide and uranyl acetate, and resiliency of the biological specimen to the electron beam and vacuum conditions as images are collected over several days^22^. Additional difficulties with tissue integrity and imaging artifacts arise with the use of complex chemical or cryofixation processes used to embed tissue blocks in plastics, limiting overall resolution^20^.

In the current work, the utility of SMLM is demonstrated for imaging FFPE tissue sections to reveal ultrastructural details of fluorescently labeled protein targets in both 2D and 3D high-resolution reconstructions. Optimized conditions for reliable retrieving and immunostaining of multiple antigens in breast cancer samples with high specificity and structural integrity were developed. SMLM was used to visualize a) HER2, a membrane receptor overexpressed in ~25% breast cancers^23^; b) TOM20, a mitochondria outer membrane protein^24^; and c) Lamin B1, a nuclear membrane protein in immuostained thin FFPE sections^25^. Results with both 2D and 3D SMLM revealed that the overall distribution of proteins and the associated cellular architecture in FFPE samples, including the cell membrane, intracellular organelles and the nuclear envelope, remain largely intact after the fixation and embedding process. In fact, many fine structural features, such as membrane protrusions that are known to be enriched in HER2 positive tissues, were clearly observed in the SMLM images of the FFPE sections. Visualization of slices of 3D reconstructions of the mitochondria, which are ~500 nm in diameter, clearly showed the expected membrane localization of TOM20. Notably, high resolution images were obtained on both 2 and 4 μm sections, suggesting that the SMLM could be integrated into existing clinical FFPE workflow. These observations demonstrate the utility of SMLM to aid in uncovering ultrastructural details of archived clinical samples and the potential value of FFPE samples in offering molecular insights into the physiopathology of tissues. By including more functionally relevant molecular markers for immunostaining and developing systematic image analysis algorithms, SMLM images of FFPE sections may help disease diagnosis and treatment as an important extension to EM-based digital pathology^26^.

## Results

### Epi-fluorescence imaging of FFPE tissue is limited

**Figure 1:**
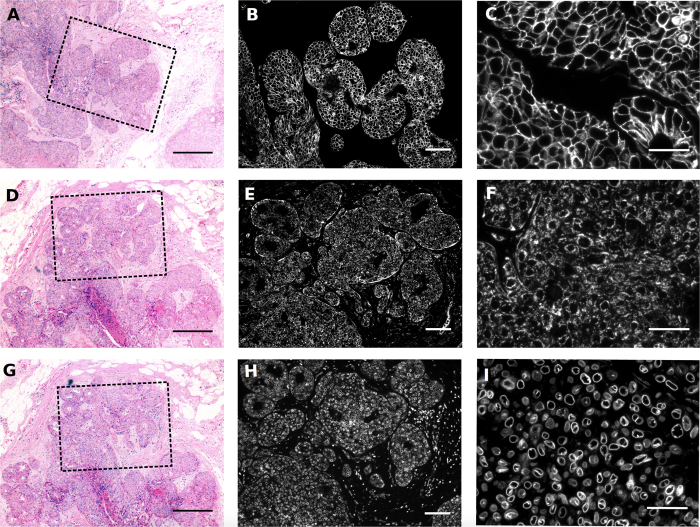
Morphology of FFPE HER2 positive, grade 3 IDC breast cancer tissue. Tissue sections were stained with H&E and overview images were collected at 5X magnification to show tumor morphology (**A**, **D** and **G**). The box in the H&E image represents the field-of-view of the adjacent immunofluorescence images. Scale bars for H&E images = 200 μm. Images of immunofluorescence staining were collected at 10X magnification to demonstrate the tissue patterns of HER2, TOM20, and Lamin B1 proteins (**B**, **E** and **H**). Scale bars = 50 μm. Additional images were collected at 40X magnification to enhance visualization of ultrastructural features of each immunostain (**C**, **F** and **I**), scale bars = 20 μm.

Conventional, epi-fluorescence microscopy was used to examine immunostained 2 and 4 μm sections of HER2+ breast cancer tissue embedded in FFPE. To evaluate the structural integrity of FFPE sections, three functional and/or structural proteins were selected as imaging targets: HER2, a receptor that is almost exclusively localized to the cell membrane in HER2+ cancers; TOM20, which labels the outer mitochondrial membrane; and Lamin B1, which marks the nuclear membrane. Whereas TOM20 and Lamin B1 label all cell types, HER2 clearly marked the cancer epithelial cells in the breast tissue. The antibody staining pattern was confirmed *in vitro* (Fig. S1) prior to staining sections of HER2+ breast cancer tissue. The ductal structure of the cancerous epithelial cells was assessed by comparing images of conventional hematoxylin and eosin (H&E) stained sections (Fig. 1A) to those of HER2 immunostained sections (Fig. 1B). However, even at high magnification, no structural features between the membranes of HER2+ cells were visible by either H&E or fluorescence on a conventional microscope (Fig. 1C). When stained with TOM20, higher mitochondrial content was seen in the cancerous epithelia cells than in the stroma (Fig. 1D,E). Similar to samples stained with HER2, however, individual mitochondria could not be visualized or assessed for structural differences even at high magnification using conventional microscopy (Fig. 1F). When stained with Lamin B1, the malignant tissues demonstrated increased cellularity and large nuclear membranes, which was confirmed using H&E staining (Fig. 1G,H). High magnification images revealed largely rounded nuclear structures, where the nuclear envelopes commonly appear as a solid line (Fig. 1I). Together, these results confirm the specific labeling of all three structural and/or functional markers in the FFPE tissue, which were used in subsequent SMLM imaging studies.

### SMLM of FFPE reveals functional ultrastructure in tissue

**Figure 2:**
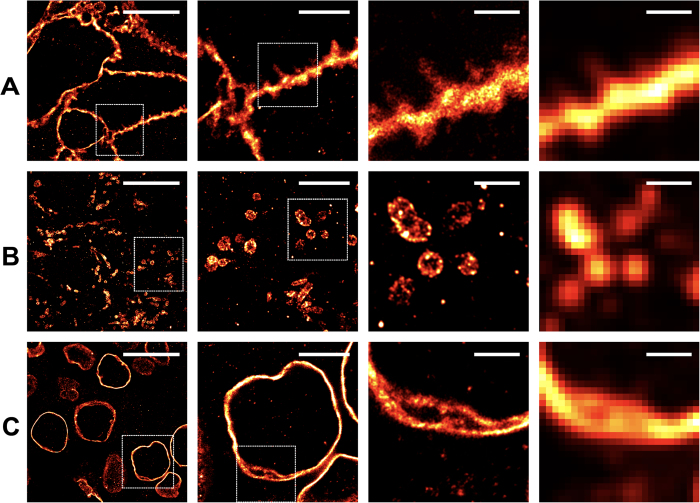
Two dimensional, super resolution imaging of HER2, TOM20, and Lamin B1 in FFPE. SMLM images at varied magnifications demonstrate the ability to resolve tissue ultrastructure not visible using conventional microscopy. Scale bars for each column of images are from left-to-right 10 μm, 4 μm, 1 μm and 1 μm, where the images in the right most column demonstrate a comparative confocal reconstruction further highlighting the necessity of SMLM to visualize nanoscopic features within the tissues. (**A)** Extracellular membrane protrusions were visible between HER2 positive cancer cells. (**B)** The outer membrane of the mitochondria and the tubular nature of this organelle were prominently resolved by SMLM imaging. (**C)** The lamin network of the nuclear lumen was highlighted.

SMLM was used to image tissue sections prepared with the same immunostaining procedures as previously described for HER2, TOM20 and Lamin B1. Enabled by both highly specific labeling of the target molecules and the superior photophysical properties of Alexa Fluor 647, raw single-molecule images in all three cases exhibited high signal-to-noise ratios (SNR, Fig. S2). SMLM images of all three molecular targets clearly exhibited distinct structural features absent from conventional epifluorescence images (Fig. 2). Fine patterns of membrane-targeted HER2, for example, were clearly visualized even in areas between adjacent epithelial cells, where protrusions between cells were readily demonstrated (Fig. 2A). Membrane protrusions enriched in HER2+ cells have been observed in cultured cells using immuno-EM^27^ and IF microscopy (Fig. S1), and these protrusions have been implicated in persistent localization and signaling of HER2 in breast cancer cells^28^. While morphologically similar protrusions in HER2+ tissues have been reported using various EM techniques^29^, it was not clear whether those were enriched in HER2. Hence, the observation of HER2-enriched protrusions in FFPE sections of breast tumors indicate that similar protrusions appear to exist *in vivo* and may play functional roles in tumor biology.

SMLM images of TOM20 and Lamin B1 also demonstrated the excellent resolving power of SMLM compared with conventional fluorescence microscopy and the well preserved structural details of both the mitochondria and the nuclear envelop in the FFPE samples (Fig. 2B,C). TOM20 images showed unambiguous localization to the mitochondria membrane (Fig. 2B). Lamin B1 images revealed additional membrane structures instead of a single, solid line marking the nuclear envelope (Fig. 2C). It is currently unclear if these structures arose from tissue cutting artifact, or are in fact novel structures not previously visualized due to resolution limitations of conventional microscopy. Additional assessment is underway to determine the source of these structural features. Assessment of the functional utility of these features will be important in future work to determine if they are related to the region of nuclear envelope sampling or may in fact be important for nuclear organization. Additionally, SMLM imaging of the nuclear envelope also demonstrated the sampling diversity in a single FFPE section where sections through the center of individual nuclei were visible as well as of the outer edges of the nuclear envelope, something not readily distinguished by conventional epi-fluorescence microscopy (Figs 1I and 2C). The tissue blocks used in these studies had been stored at room temperature for 11 years prior to the staining and imaging experiments. The acquired images demonstrated the ability to preserve antigenicity after room temperature storage for more than a decade, similar to previous work using STED microscopy^19^.

### SMLM enables 3D visualization of functional ultrastructure

**Figure 3:**
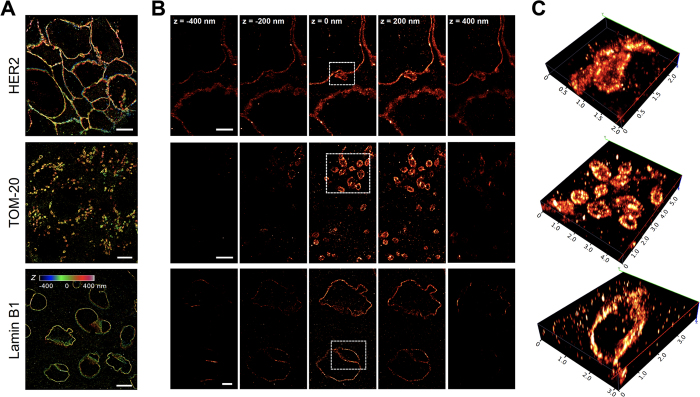
Imaging HER2+ tumor FFPE sections with 3D SMLM. (**A)** Reconstructed 3D SMLM images of FFPE sections immunostained for HER2 (top), TOM-20 (middle), and Lamin-B1 (bottom) with Z-positions of the molecules color coded. (**B)** Slice views of 3D SMLM images of HER2 (top), TOM-20 (middle), and Lamin-B1 (bottom) in 200 nm increments are demonstrated where each slice represents the projections of all localization events within 20 nm relative to the z-plane. All images were collected on 2 μm sections. (**C)** Volumetric rendering of the boxed areas in B are shown. Scale bars as follows: 10 μm (**A**), 2 μm (**B**, top and bottom), 1 μm (**B**, middle).

Conventional FFPE sections are relatively thick by high resolution microscopy standards, where slice thickness for pathological analysis is usually 4 μm^30^. Imaging 4 μm in depth using SMLM is challenging as typical depth of focus is ~800 nm^31^. To enable 3D imaging of these sections, a cylindrical lens was inserted in the detection path allowing encoding of z-position into eccentricity of the point spread functions (PSFs) (Fig. 3). To facilitate use of gold nanoparticles as fiducial markers, the focus was typically set at 200–400 nm above the coverslip so the gold particles were well within the image depth. In this setting, we estimated that the lateral (x-, y-) resolution was on the order of 20–40 nm and the axial (z-) resolution was around 60–80 nm (Fig. S3). The resolution achieved in all three dimensions were slightly lower than previous reports^15^,^31^ or those achieved on culture cells using similar setups, likely due to the slightly elevated background in the single-molecule images and potential mechanical instability of the sections on the coverslip.

Compared with the 2D images, which were projections of features within the entire slab, imaging in 3D revealed even greater ultrastructural features within much finer (~100 nm) optical sections above the coverslip (Fig. 3B). For example, membrane vesicles (blebs) with HER2 staining were clearly visible, as previously demonstrated using stimulated emission depletion (STED) imaging of FFPE tissues^19^. Three dimensional imaging also facilitated assessment of mitochondrial arrangement within the cytosol surrounding the nuclei in malignant cells in all directions. Nuclear structure was also more readily assessed where the top and bottom of the structures were easily visualized as well as folds in the membrane structure that appeared to be sample artifact in 2D imaging studies (Figs 2 and 3).

### Impact of sample thickness on image quality

**Figure 4:**
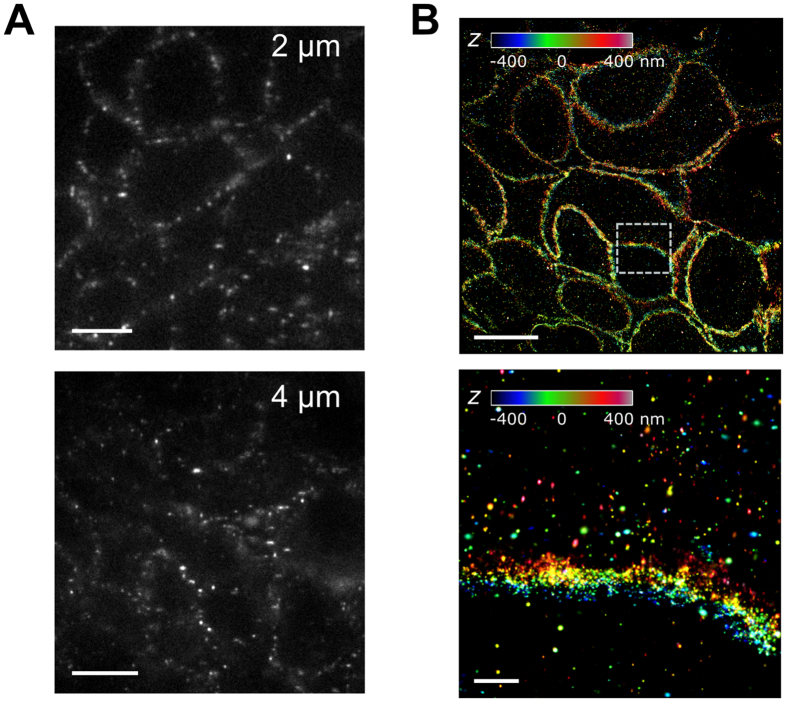
Comparing 2 and 4 μm FFPE sections for 3D SMLM imaging. (**A)** Representative astigmatic single-molecule images collected on 2 and 4 μm sections. (**B)** Reconstructed 3D SMLM image of a 4 μm FFPE tumor section immunolabeled for HER2, with z-positions color coded (see legend). The bottom image shows the zoomed-in view of the boxed area in the top image. Scale bars, 5 μm (left), 10 μm (right, top) and 2 μm (right, bottom).

Localization precision and hence resolution of SMLM is sensitive to background, which can increase with sample thickness. To assess the effect of slice thickness on imaging resolution, SMLM images on 2 and 4 μm FFPE sections stained for HER2 were compared with respect to background in raw, single-molecule images as well as the quality of reconstructed, high-resolution images. Despite the increased thickness, raw single-molecule images taken on 4 μm sections were of similar quality to those collected on 2 μm sections, both by visual inspection (Fig. 4A) and by analyzing the signal-to-noise ratios (Figs S2 and S4C) and goodness of fit of individual single-molecule images (data not shown). Surprisingly, however, in the reconstructed SMLM images of 4 μm sections, there were visibly higher densities of cytosolic HER2 signals in SMLM images of 4 μm sections (Fig. 4B) compared with those reconstructed from data collected on 2 μm section (Figs 2 and 3). By contrast, HER2 was predominantly membrane-bound in cultured SKBR3 cells (Fig. S1 and unpublished data). Hence, we considered the cytosolic HER2 signals in Fig. 4B to be background or nonspecific staining. A quantitative comparison of background staining for 2 versus 4 μm sections was completed (Fig. S4), from which it was evident that the thicker section presented higher background. However, the background difference was <2-fold, with the pixel areas covered by background representing ~4% to ~7% for 2 and 4 μm sections, respectively, both with large spatial variations. The increased background in thicker sections could be due in part to less robust antigen retrieval and immunostaining for thicker tissue section, as some previous reports have suggested^32^. Despite the increased background, signal (~100% pixel coverage on the membrane) to background (4–7% pixel coverage in the cytosol) ratio remained high such that it would not prevent practical use of SMLM to image 4 μm thick tissue specimens. That said, the increased background may become a limiting factor if much thicker samples were desired for SMLM imaging. On a practical note, it was much easier to prepare 4 μm sections without tissue damage or cutting artifact. In part for this reason, 4 μm tissue sections are more commonly used in the clinical settings. Thus, the ability to image 4 μm thick FFPE sections with SMLM provides a potential link to clinically relevant sample handling procedures, where the samples can be imaged both by SMLM for nondestructive characterization of tissue ultrastructure and by conventional pathological analyses such as IF and/or H&E.

## Discussion

Herein, we have demonstrated the application of SMLM on FFPE sections milled at traditional histological thickness facilitating generation of detailed, spatial-molecular data at nanometer resolution. FFPE tissues have been used by clinical pathology for almost a century and today, millions of FFPE samples are stored in bio-repositories worldwide. The developed SMLM imaging methodology adds to the arsenal of measurement technologies utilized by modern precision medicine to characterize proteomic and genomic signatures in health and disease. In particular, this novel SMLM methodology permits detailed investigation of tissue architecture and molecular interactions in clinical specimens at nanometer resolution, generating data concomitant to technological standards such as H&E, IF, EM, and gene expression microarrays.

SMLM affords simultaneous ultrastructural and histochemical analysis of FFPE samples at 10–50 nm resolution while maintaining full compatibility with existing histopathological sample preparation and tissue staining protocols. Routine histopathology relies on the ubiquitous, low-cost H&E stained FFPE specimens with the addition of ancillary methods such as flow-cytometry, IHC/IF stains and *in situ* hybridization to uncover etiologic data^33^. EM is employed in this setting to visualize complex ultrastructures and abnormalities invisible to conventional light microscopy, and it plays a diagnostic role in assessing tumor differentiation^33^. However, depending on the EM technique utilized, unique preservatives, section thickness, and embedding materials not compatible with FFPE are required to attain accurate information from the tissue specimen. Compared with EM, simplicity in sample preparation is an immediate advantage of the developed SMLM methodology, as serial sections from routine FFPE blocks can be prepared for SMLM with only minor adjustments to the IF staining protocol, primarily in the concentration and class of utilized fluorophores. SMLM also provides more specific and generally higher efficiency labeling than either the Tokuyasu technique or other post-embedding immunostaining methods used for EM^34^. Additionally, samples prepared for SMLM imaging can be imaged using conventional microscopy without additional staining or sample preparation steps, enabling convenient association between high resolution SMLM structures to those visualized in histopathology (Fig. 1), which is not the case for samples prepared for EM.

Although fluorescence imaging of tissue sections has been completed using both confocal and superresolution microscopies^17^,^18^,^19^,^35^,^36^, results with SMLM presented here are improved in many respects. The spatial resolution obtained here on FFPE sections with SMLM is ~30 nm laterally and ~70 nm axially, which is on par with previous work with STED^19^ and clearly better than conventional confocal microscopy (Figs 1 and 2). Both STED and SMLM offer the potential for 3D resolving power, where 3D SMLM FFPE imaging has been demonstrated herein and offers a powerful alternative to STED for analyzing tissue ultrastructures in archived samples. The earliest attempt to use SMLM to resolve tissue ultrastructure was achieved by Dani *et al*. in 2010 to resolve dense protein organizations at the synaptic cleft of frozen brain specimens with 3D astigmatism imaging^17^. While native antigenicity was better preserved in fresh frozen sections for immunostaining compared to FFPE preserved tissues, frozen tissue preservation came at the cost of diminished tissue ultrastructure preservation. The limitations of using SMLM in frozen sections to characterize cellular ultrastructure were well demonstrated by Dani *et al*., and the importance of developing a superresolution platform for visualizing organelle markers with membrane contrast comparable to EM was highlighted^17^.

The superior spatial resolution and ultrastructure preservation using SMLM imaging in FFPE tissue were demonstrated herein, where features not accessible through conventional fluorescence microscopy became visible using SMLM (Figs 2 and 3). Undulations in the nuclear membrane and individual mitochondrial membranes were both clearly shown in the SMLM images of FFPE sections (Fig. 2B,C). Additionally, imaging of labeled HER2 in the outer membrane demonstrated exquisite resolution of the ultrastructure and new nanoscopic features in tissue, specifically membrane protrusions (Fig. 2A), which had only previously been observed in high-resolution *in vitro* imaging experiments (Fig. S1) or with EM^27^. We also observed vesicle-like structures decorated with HER2, similar to those previously reported by Ilgen *et al*. in 2014 using STED microcopy on HER2-positive rectal adenocarcinoma FFPE specimens^19^. Further SMLM imaging of additional biomarkers will surely enable identification of nanoscopic structures not previously associated with specific proteins.

Importantly, SMLM is fully compatible with existing FFPE sample processing and imaging procedures as sections cut at standard thickness (typically 4 μm) can be directly imaged (Fig. 4). To maximize resolution and signal collection efficiency, SMLM typically uses high numerical-aperture objectives that have shallow focus depth. This limits the range of z depth for a SMLM to ~800 nm or less, and additional sample volume beyond this range can add to the background. Much to our surprise, the image quality and nanoscopic resolution using 2 and 4 μm sections were comparable despite the slightly elevated background in the 4 μm sections (Figs S2 and S4C). This is a significant finding as it will enable SMLM integration into conventional clinical workflow using FFPE sections cut at 4 μm thickness to reveal 3D architectural details. Furthermore, as demonstrated in previous reports, thick biological samples such as whole cells can be imaged with 3D SMLM by using optical sectioning, where each optical section is ~800 nm or less^15^. Hence, it is possible to image through the entire 4 μm section with at least ~30 nm lateral and ~70 nm axial resolutions in approximately 5 to 10 optical sections. Although the resolution is not as high as volumetric EM imaging^20^, SMLM does not require physical sectioning and hence is free of sectioning artifacts or challenges in registering images from multiple physical sections.

In summary, the work presented here demonstrates the possibility of using SMLM to image FFPE tissues with high resolution in all three dimensions. Three functional and structural markers of breast cancer were demonstrated to translate from IF imaging using conventional microscopy to nanoscopic visualization through SMLM. The high resolution afforded by SMLM facilitated visualization of structures not resolvable by conventional fluorescence microscopy including HER2 enriched membrane protrusions, membrane vesicles, mitochondrial membranes, and folds in the nuclear membrane. These features as well as yet undiscovered nanoscopic structures could play a vital role in disease diagnosis and treatment and offer critical insights for mechanistic understanding of disease and therapeutic response. Importantly, SMLM sample preparation is compatible with current pathological sample preparation, and hence SMLM can be readily integrated into current clinical workflow. Given the vast repository of FFPE blocks and the rich information associated with these samples, our work extends imaging of FFPE to the nanoscopic scale and opens up unique opportunities for exploring tissue ultra- and nano-structures in disease diagnosis, progression and treatment.

## Methods

### Tissue Selection and Sectioning

Formalin-fixed paraffin-embedded (FFPE) primary, human stage three infiltrating ductal carcinoma (IDC) tissue that had been stored as a formalin fixed paraffin embedded block for 11 years was obtained from the Oregon Health and Science University (OHSU) Knight Tissue Bank (Portland, OR). The tissue samples were acquired and processed under institutional review board (IRB) approval for the OHSU Knight Tissue Bank. Tissue used in all experiments was surgically resected in 2004 and was prescreened for breast cancer grade, stage and hormone receptor status including HER2, estrogen receptor (ER) and progesterone receptor (PR). Tissues selected for this study had high HER2 expression denoted as +3 with robust membrane staining and minimal necrosis (<25%) where the majority of the tissue block contained tumor tissue (>50%). FFPE blocks were cooled to −5 °C on ice blocks and serially sectioned using a microtome (RM2125 RTS, Leica Biosystems, Germany) at 2 or 4 μm thickness. Sections were mounted onto charged glass slides (Superfrost Plus Slides, Thermo Fisher Scientific, Carlsbad, CA) and baked in a drying oven at 52 °C for 30 minutes. Tissue staining by H&E and immunofluorescence were completed on consecutive sections.

### Cell Culture

SKBR3 cells were cultured at 37 °C and 5% CO_2_ in McCoy’s 5 A media (Thermo Fisher Scientific), supplemented with 10% fetal bovine serum (HyClone Labs, GE Healthcare, Chalfont, UK) and 1% Penicillin/Steptomycin-Glutamine (Thermo Fisher Scientific). Cells were plated in 96-well plates with #1.5 coverglass bottoms (*In Vitro* Technologies, Australia) for conventional fluorescence microscopy. For superresolution imaging, 8-well chamber plates with #1.5 coverglass bottoms (Lab-Tek II, Thermo Fisher Scientific) were cleaned using NaOH (1 M) for two hours, washed with PBS (3 × 15 min) and left in fresh PBS overnight. Cells were platted at 1 × 10^5^ cells per well and incubated for 3 days to reach about 50% confluence for imaging.

### Labeling Secondary Antibodies

Donkey anti-rabbit secondary antibody (Jackson ImmunoResearch, West Grove, CA) was conjugated to Alexa Fluor 647 (AF647) succinimidyl ester (Thermo Fisher Scientific) using previously published methods^37^. Briefly, the antibody was washed in three changes of 1× PBS using a 10 K molecular weight cut off (MWCO) spin column (Vivaspin, Thermo Fisher Scientific) followed by volume adjusted of the washed antibody to 1 mL of PBS (pH 7.4). Fifteen molar equivalents of AF647 succinimidyl ester were added and the reaction was agitated at room temperature for 3 hours. Medium pressure chromatography (NGC Quest 10 Plus w/mutli-wavelength [UV/Vis] detection, Bio-Rad, Hercules, CA) was used to separate the conjugated antibody from unbound fluorophore using a desalting column (Bio-Scale Mini Bio-Gel P-6 Cartridge, Bio-Rad). The purified product was concentrated using 10 K MWCO spin filters to a final antibody concentration of 5.0 μM in 200 μL of PBS. Fluorophore and protein concentrations were calculated from absorbance measurements (NanoDrop 2000c, Thermo Fisher Scientific) and used to calculate the final fluorophore to antibody ratio which was ~1.5 for all secondary antibody conjugates.

### Automated Hematoxylin and Eosin (H&E) Staining

An Autostainer XL (ST5010, Leica Biosystems, Nussloch, Germany) was used to perform standard H&E staining. The automated staining protocol consisted of the following steps: two changes of xylenes (1 min each), rehydration in an ethanol/water gradient (100%, 95%, and 70% ethanol for 1 min each), followed by washing with deionized (DI) water (1 min). The rehydrated slides were stained with Gill 2 Hematoxylin (5 min, Thermo Fisher Scientific), washed with DI water (90 sec), followed by a brief wash with Nu-Clear II (20 sec, Thermo Fisher Scientific), a subsequent wash with DI water (90 sec), and a final incubation with Bluing Reagent (1 min, Thermo Fisher Scientific). Staining with Eosin Y (0.25% in ethanol, 30 sec, Thermo Fisher Scientific) was completed, followed by three washes with 100% ethanol (1 min each), and three washes with xylenes (1 min each). Lastly, H&E stained slides were cover slipped with Permount (Thermo Fisher Scientific) and allowed to dry flat prior to imaging.

### Immunofluorescence Staining

Cultured cells were fixed with 3.7% Paraformaldehyde (PFA) in 1× PHEM buffer (120 mM PIPES, 50 mM HEPES, 19 mM EGTA, 16 mM MgSO_4_, pH 7.0) for 20 minutes followed by washing with 1× PBS (3 × 5 min). Cells were permeabilized and blocked in a mixture of 3% bovine serum albumin (BSA) and 0.5% Triton X-100 in 1× PBS at pH 7.4 for 30 minutes. Primary antibodies HER2 (1:300, Ab134182, Abcam, Cambridge, UK), TOMM20 (1:500, Ab78547, Abcam), and Lamin B1 (1:250, Ab65986, Abcam) were applied to individual samples and incubated for 40 minutes at room temperature. Following removal of the primary antibody, cells were washed in 1× PBS (3 × 5 min). AF647 conjugated goat anti-rabbit secondary antibody was incubated with the cells at a protein concentration of 0.1 mM for 30 minutes protected from light. The secondary antibody was removed and the cells were washed with 1× PBS (3 × 5 min). The stained cells were post-fixed with 3.7% PFA in 1× PHEM buffer for 10 minutes. Following fixation, the cells were washed with 1× PBS and stored in the dark until imaging. For SMLM imaging, 150 nm gold colloid (8.5 × 10^7^ particles/mL, Ted Pella, Redding, CA) was suspended in DPBS with Ca^2+^/Mg^2+^ and applied to the stained cells 30 minutes prior to imaging.

FFPE sections were deparaffinized in xylenes (2 × 10 min) and rehydrated in an ethanol/water gradient series: 100% (2 × 10 min), 95% (5 min), 70% (5 min), and 50% (5 min) ethanol, respectively. The rehydrated slides were briefly immersed in water and then washed in 1× PBS (10 min). Epitopes were unmasked using a two-step antigen retrieval technique modified from Gerdes *et al*.^38^ where staining buckets containing Tris buffer (10 mM Tris Base, 0.05% Tween 20, pH 8.0) and Citrate buffer (10 mM Sodium Citrate, 0.05% Tween 20, pH 6.0) were pre-heated simultaneously in a pressure cooker containing 1 L of DI water. Slides were pressure-cooked for 20 minutes on the highest setting in Tris buffer. Slides were then transferred to the heated Citrate buffer and cooled to room temperature (45 minutes). Slides were washed once in 0.1% Tween-20 (5 min) and once in PBS (5 min). The tissue sections were permeabilized with 0.4% Triton X-100 in PBS for 45 minutes, washed in PBS (2 × 5 min) and blocked with 3% BSA for 1 hour. Slides were blotted to remove the BSA and the primary antibodies were applied at predetermined concentrations as follows: rabbit monoclonal to HER2 (1:100), rabbit polyclonal to TOMM20 (1:50), and rabbit polyclonal to Lamin B1 (1:100). Slides were incubated overnight at 4 °C in a humidified chamber and then washed with PBS (3 × 5 min). AF647 conjugated anti-rabbit secondary antibodies (711–005–152, Jackson ImmuoResearch) were applied to the tissue sections at a protein concentration of 0.3 mM and incubated in the dark at room temperature for one hour. Slides were washed in PBS (3 × 5 min), fixed with 3.7% PFA for 10 minutes, and again washed with PBS (2 × 5 min). The slides were cover slipped (22 × 30 mm, no. 1.5, VWR micro cover glass), sealed with clear nail varnish and stored in the dark at −20 °C prior to imaging. Fluoromount-G (Southern Biotech, Birmingham, AL) was used as mounting media for standard fluorescent imaging. For SMLM imaging, fresh imaging buffer was made from stock components (see SMLM imaging buffer preparation section below), applied to the sample and cover slipped 5 minutes before imaging.

### Light and Epi-Fluorescence Microscopy

H&E-stained and standard immunofluorescence samples were imaged using a Zeiss Axio Observer inverted microscope (Zeiss, Oberkochen, Germany). A metal halide light source (PhotoFluor II NIR, 89 North, Burlington, VT) was used for phase contrast and filtered for appropriate fluorescence excitation using a 620 ± 30 nm bandpass excitation filter (Chroma Technologies, Bellows Falls, VT). AF647 fluorescence emission was imaged using a 700 ± 38 nm bandpass emission filter (Chroma Technologies). Images were collected with an Axiocam 506 monochrome camera (Zeiss) and an Axiocam 105 color camera (Zeiss). Representative images of tissue staining for each target were collected using 5×, 10×, 20×, and 40× objectives. For cell culture, representative images were collected using 40× and 63× objectives. Images were collected with optimal exposure times that ranged from 500–5000 msec. Serially sectioned FFPE tissue were imaged with the aligned monochrome and color cameras enabling the same H&E regions of interest (ROIs) to be imaged as those on the corresponding immunofluorescence stained section.

### SMLM Imaging Buffer Preparation

The imaging buffer was prepared similarly to previously described methods^31^, by mixing a Tris Normal (TN) buffer, GLOX, and MEA (β-mercaptoethylamine). In brief, the TN buffer was made by dissolving 10% glucose (w/v, Fisher Chemicals D16–500) in 50 mM Tris buffer with 10 mM NaCl (pH 8.0) where prepared TN buffer was stored at 4 °C for up to several months. The GLOX stock solution contained 0.5 mg/mL glucose oxidase (Sigma-Aldrich, G2133–50 kU) and 40 μg/mL catalase (Sigma-Aldrich, C100-50MG). The GLOX mixture was centrifuged briefly at ~3000 × g to remove any precipitates prior to use. MEA (Sigma-Aldrich, 30070) was dissolved at 1 M in 0.25 mM hydrogen chloride (HCl) and stored at 4 °C for a maximum of two weeks. Prior to each experiment, the imaging buffer was prepared fresh by mixing GLOX, MEA, and TN at a ratio of 1:1:98.

### SMLM Instrumentation, Image Acquisition and Analysis

SMLM measurements were performed on a custom PALM/STORM setup constructed on a Nikon Ti-U inverted microscope frame equipped with an oil immersion objective (APON, 60XOTIRF, NA 1.49, Olympus, effective magnification ~66× on a Nikon microscope body), two lasers emitting at 647 nm (OBIS, Coherent) and 405 nm (CUBE, Coherent), respectively, and an EM-CCD camera (Evolve 512 Delta, Photometrics). Light from the two lasers was expanded, combined, and projected onto the back aperture of the objective through a focusing lens (f = 400 mm) to achieve wide field and total internal reflectance (TIRF) illumination. Fluorescence signals were collected through the same objective, separated from the lasers with a quad-edge beamsplitter (Di01-R405/488/561/635, Semrock), and filtered using a band pass filter (FF01-697/75, Semrock) before being imaged on the EM-CCD. The 1.5× tube lens was selected to achieve a total 100× magnification. A weak cylindrical lens (f~1000 mm) was placed in front of the EM-CCD to generate astigmatism for 3D localization.

For SMLM imaging of AF647, both the 647 nm laser (1–2 kW/cm^2^) and the 405 nm laser (1–10 W/cm^2^) were applied to the sample simultaneously. Mechanical shutters individually controlled the two lasers, and the intensity of the 405 nm laser was increased gradually as the imaging progressed to ensure appropriate switching rates of the AF647 fluorophores. At these power densities, images were acquired at 50–70 Hz, and each data set (SMLM ‘movie’) contained 20,000 to 50,000 frames. A custom-built focus stabilization system was used in all experiments to maintain the image focus within ±25 nm. All image acquisition was done with micro-manager^39^.

Raw SMLM movies were processed using both ThunderSTORM (for 3D data sets)^40^ and a custom-written MatLab package (wfiread and palm for 2D data sets)^41^. For 3D data processing, a calibration curve was generated using images of 40 nm fluorescence beads scattered on a coverslip taken at a series of focal positions, and the resulting calibration file was imported into ThunderSTORM to convert the measured widths of individual single molecule images into axial coordinates. Color-coded 3D SMLM images were subsequently generated using ImageJ. Processing of 2D data sets using the wfiread and palm software packages have been previously described^41^.

## Additional Information

**How to cite this article**: Creech, M. K. *et al*. Superresolution Imaging of Clinical Formalin Fixed Paraffin Embedded Breast Cancer with Single Molecule Localization Microscopy. *Sci. Rep.* **7**, 40766; doi: 10.1038/srep40766 (2017).

**Publisher's note:** Springer Nature remains neutral with regard to jurisdictional claims in published maps and institutional affiliations.

## Supplementary Material

Supplementary Information

## References

[b1] Baker M. Biorepositories: Building better biobanks. Nature 486, 141–146 (2012). 22678297 10.1038/486141a

[b2] Cejas P. . Chromatin immunoprecipitation from fixed clinical tissues reveals tumor-specific enhancer profiles. Nat Med (2016). 10.1038/nm.4085 27111282

[b3] Lucena-Aguilar G. . DNA Source Selection for Downstream Applications Based on DNA Quality Indicators Analysis. Biopreserv Biobank 1–7 (2016). 27158753 10.1089/bio.2015.0064 PMC4991598

[b4] Oh E. . Comparison of Accuracy of Whole-Exome Sequencing with Formalin-Fixed Paraffin-Embedded and Fresh Frozen Tissue Samples. PLoS One 10, e0144162 (2015). 26641479 10.1371/journal.pone.0144162 PMC4671711

[b5] Najarian R. . Primary structure and gene organization of human hepatitis A virus. Proc Natl Acad Sci USA 82, 2627–2631 (1985). 2986127 10.1073/pnas.82.9.2627 PMC397617

[b6] Lou J. J. . A review of room temperature storage of biospecimen tissue and nucleic acids for anatomic pathology laboratories and biorepositories. Clin Biochem 47, 267–273 (2014). 24362270 10.1016/j.clinbiochem.2013.12.011 PMC3976177

[b7] Kokkat T. J., Patel M. S., McGarvey D., LiVolsi V. A. & Baloch Z. W. Archived formalin-fixed paraffin-embedded (FFPE) blocks: A valuable underexploited resource for extraction of DNA, RNA, and protein. Biopreserv Biobank 11, 101–106 (2013). 24845430 10.1089/bio.2012.0052 PMC4077003

[b8] Gerlach J. v. Mikroskopische Studien aus dem Gebeite der menschlichen Morphologie. (Erlangen: Ferdinand Enke., 1858).

[b9] Weiss S. Shattering the diffraction limit of light: a revolution in fluorescence microscopy? Proc Natl Acad Sci USA 97, 8747–8749 (2000). 10922028 10.1073/pnas.97.16.8747 PMC34005

[b10] Baday M. . Multicolor super-resolution DNA imaging for genetic analysis. Nano Lett 12, 3861–3866 (2012). 22698062 10.1021/nl302069q PMC3880789

[b11] Pereira C. F., Rossy J., Owen D. M., Mak J. & Gaus K. HIV taken by STORM: super-resolution fluorescence microscopy of a viral infection. Virol J 9, 84 (2012). 22551453 10.1186/1743-422X-9-84 PMC3409066

[b12] Ptacin J. L. . A spindle-like apparatus guides bacterial chromosome segregation. Nat Cell Biol 12, 791–798 (2010). 20657594 10.1038/ncb2083 PMC3205914

[b13] Sochacki K. A. . Imaging the post-fusion release and capture of a vesicle membrane protein. Nat Commun 3, 1154 (2012). 23093191 10.1038/ncomms2158 PMC3521636

[b14] Huang B., Babcock H. & Zhuang X. Breaking the diffraction barrier: super-resolution imaging of cells. Cell 143, 1047–1058 (2010). 21168201 10.1016/j.cell.2010.12.002 PMC3272504

[b15] Huang B., Jones S. A., Brandenburg B. & Zhuang X. Whole-cell 3D STORM reveals interactions between cellular structures with nanometer-scale resolution. Nat Methods 5, 1047–1052 (2008). 19029906 10.1038/nmeth.1274 PMC2596623

[b16] Jones S. A., Shim S. H., He J. & Zhuang X. Fast, three-dimensional super-resolution imaging of live cells. Nat Methods 8, 499–508 (2011). 21552254 10.1038/nmeth.1605 PMC3137767

[b17] Dani A., Huang B., Bergan J., Dulac C. & Zhuang X. Superresolution imaging of chemical synapses in the brain. Neuron 68, 843–856 (2010). 21144999 10.1016/j.neuron.2010.11.021 PMC3057101

[b18] Sams M. . Spatial cluster analysis of nanoscopically mapped serotonin receptors for classification of fixed brain tissue. J Biomed Opt 19, 011021 (2014). 24297043 10.1117/1.JBO.19.1.011021

[b19] Ilgen P. . STED super-resolution microscopy of clinical paraffin-embedded human rectal cancer tissue. PLoS One 9, e101563 (2014). 25025184 10.1371/journal.pone.0101563 PMC4099123

[b20] Peddie C. J. & Collinson L. M. Exploring the third dimension: volume electron microscopy comes of age. Micron 61, 9–19 (2014). 24792442 10.1016/j.micron.2014.01.009

[b21] Pinali C. & Kitmitto A. Serial block face scanning electron microscopy for the study of cardiac muscle ultrastructure at nanoscale resolutions. J Mol Cell Cardiol 76, 1–11 (2014). 25149127 10.1016/j.yjmcc.2014.08.010

[b22] Helmstaedter M. . Connectomic reconstruction of the inner plexiform layer in the mouse retina. Nature 500, 168–174 (2013). 23925239 10.1038/nature12346

[b23] Iqbal N. & Iqbal N. Human Epidermal Growth Factor Receptor 2 (HER2) in Cancers: Overexpression and Therapeutic Implications. Mol Biol Int 2014, 852748 (2014). 25276427 10.1155/2014/852748 PMC4170925

[b24] Eliyahu E. . Tom20 mediates localization of mRNAs to mitochondria in a translation-dependent manner. Mol Cell Biol 30, 284–294 (2010). 19858288 10.1128/MCB.00651-09 PMC2798288

[b25] Burke B. & Stewart C. L. The nuclear lamins: flexibility in function. Nat Rev Mol Cell Biol 14, 13–24 (2013). 23212477 10.1038/nrm3488

[b26] Pantanowitz L. Digital images and the future of digital pathology. J Pathol Inform 1 (2010). 10.4103/2153-3539.68332 PMC2941968 20922032

[b27] Hommelgaard A. M., Lerdrup M. & van Deurs B. Association with Membrane Protrusions Makes ErbB2 an Internalization-resistant Receptor. Molecular Biology of the Cell 15, 1557–1567 (2004). 14742716 10.1091/mbc.E03-08-0596 PMC379255

[b28] Jeong J. . PMCA2 regulates HER2 protein kinase localization and signaling and promotes HER2-mediated breast cancer. Proc Natl Acad Sci USA 113, E282–290 (2016). 26729871 10.1073/pnas.1516138113 PMC4725473

[b29] Hommelgaard A. M., Lerdrup M. & van Deurs B. Association with membrane protrusions makes ErbB2 an internalization-resistant receptor. Mol Biol Cell 15, 1557–1567 (2004). 14742716 10.1091/mbc.E03-08-0596 PMC379255

[b30] Bass B. P., Engel K. B., Greytak S. R. & Moore H. M. A review of preanalytical factors affecting molecular, protein, and morphological analysis of formalin-fixed, paraffin-embedded (FFPE) tissue: how well do you know your FFPE specimen? Arch Pathol Lab Med 138, 1520–1530 (2014). 25357115 10.5858/arpa.2013-0691-RA

[b31] Huang B., Wang W., Bates M. & Zhuang X. Three-Dimensional Super-Resolution Imaging by Stochastic Optical Reconstruction Microscopy. Science 319, 810–813 (2008). 18174397 10.1126/science.1153529 PMC2633023

[b32] Boenisch T. Pretreatment for immunohistochemical staining simplified. Appl Immunohistochem Mol Morphol 15, 208–212 (2007). 17525636 10.1097/01.pai.0000209862.28205.e6

[b33] Makki J. S. Diagnostic Implication and Clinical Relevance of Ancillary Techniques in Clinical Pathology Practice. Clin Med Insights Pathol 9, 5–11 (2016). 27042154 10.4137/CPath.S32784 PMC4807883

[b34] Bos E. . Vitrification of Tokuyasu-style immuno-labelled sections for correlative cryo light microscopy and cryo electron tomography. J Struct Biol 186, 273–282 (2014). 24704216 10.1016/j.jsb.2014.03.021

[b35] Schedin-Weiss S., Caesar I., Winblad B., Blom H. & Tjernberg L. O. Super-resolution microscopy reveals gamma-secretase at both sides of the neuronal synapse. Acta Neuropathol Commun 4, 29 (2016). 27036709 10.1186/s40478-016-0296-5 PMC4818506

[b36] Schoen M. . Super-Resolution Microscopy Reveals Presynaptic Localization of the ALS/FTD Related Protein FUS in Hippocampal Neurons. Front Cell Neurosci 9, 496 (2015). 26834559 10.3389/fncel.2015.00496 PMC4709451

[b37] Gibbs S. L. . Near-infrared fluorescent digital pathology for the automation of disease diagnosis and biomarker assessment. Mol Imaging 14 (2015). 10.2310/7290.2015.00005 PMC4384048 25812603

[b38] Gerdes M. J. . Highly multiplexed single-cell analysis of formalin-fixed, paraffin-embedded cancer tissue. Proc Natl Acad Sci USA 110, 11982–11987 (2013). 23818604 10.1073/pnas.1300136110 PMC3718135

[b39] Edelstein A. D. . Advanced methods of microscope control using muManager software. J Biol Methods 1 (2014). 10.14440/jbm.2014.36 PMC4297649 25606571

[b40] Ovesny M., Krizek P., Borkovec J., Svindrych Z. & Hagen G. M. ThunderSTORM: a comprehensive ImageJ plug-in for PALM and STORM data analysis and super-resolution imaging. Bioinformatics 30, 2389–2390 (2014). 24771516 10.1093/bioinformatics/btu202 PMC4207427

[b41] Nickerson A., Huang T., Lin L. J. & Nan X. Photoactivated Localization Microscopy with Bimolecular Fluorescence Complementation (BiFC-PALM). J Vis Exp, e53154 (2015). 26779930 10.3791/53154 PMC4758764

