# Supplementary material for: Superresolution Imaging of Clinical Formalin Fixed Paraffin Embedded Breast Cancer with Single Molecule Localization Microscopy
Source: Sci Rep. 2017 Jan 18;7:40766. doi: 10.1038/srep40766 (PMC5241681; doi:10.1038/srep40766)
Supplement: Supplementary Information [file srep40766-s1.pdf]

Supplementary Information

Superresolution Imaging of Clinical Formalin Fixed Paraffin Embedded Breast Cancer with Single Molecule Localization Microscopy

Matthew K. Creech[a*], Jing Wang[a*], Xiaolin Nan[a,b,c,§] Summer L. Gibbs[a,b,c,§]

[a]Biomedical Engineering Department, [b]Knight Cancer Institute, [c]OHSU Center for Spatial Systems Biomedicine, Oregon Health & Science University, Portland, OR 97201

[*]These authors contributed equally to this work.

[§]Corresponding Authors:

Xiaolin Nan, Ph.D.
Oregon Health & Science University
Collaborative Life Sciences Building
2730 SW Moody Ave, Mail Code: CL3N
Portland, OR 97201
Email: nan@ohsu.edu
Phone: 503-418-9317

Summer L. Gibbs, Ph.D.
Oregon Health & Science University
Collaborative Life Sciences Building
2730 SW Moody Ave, Mail Code: CL3SG
Portland, OR 97201
Email: gibbss@ohsu.edu
Phone: 503-494-8940

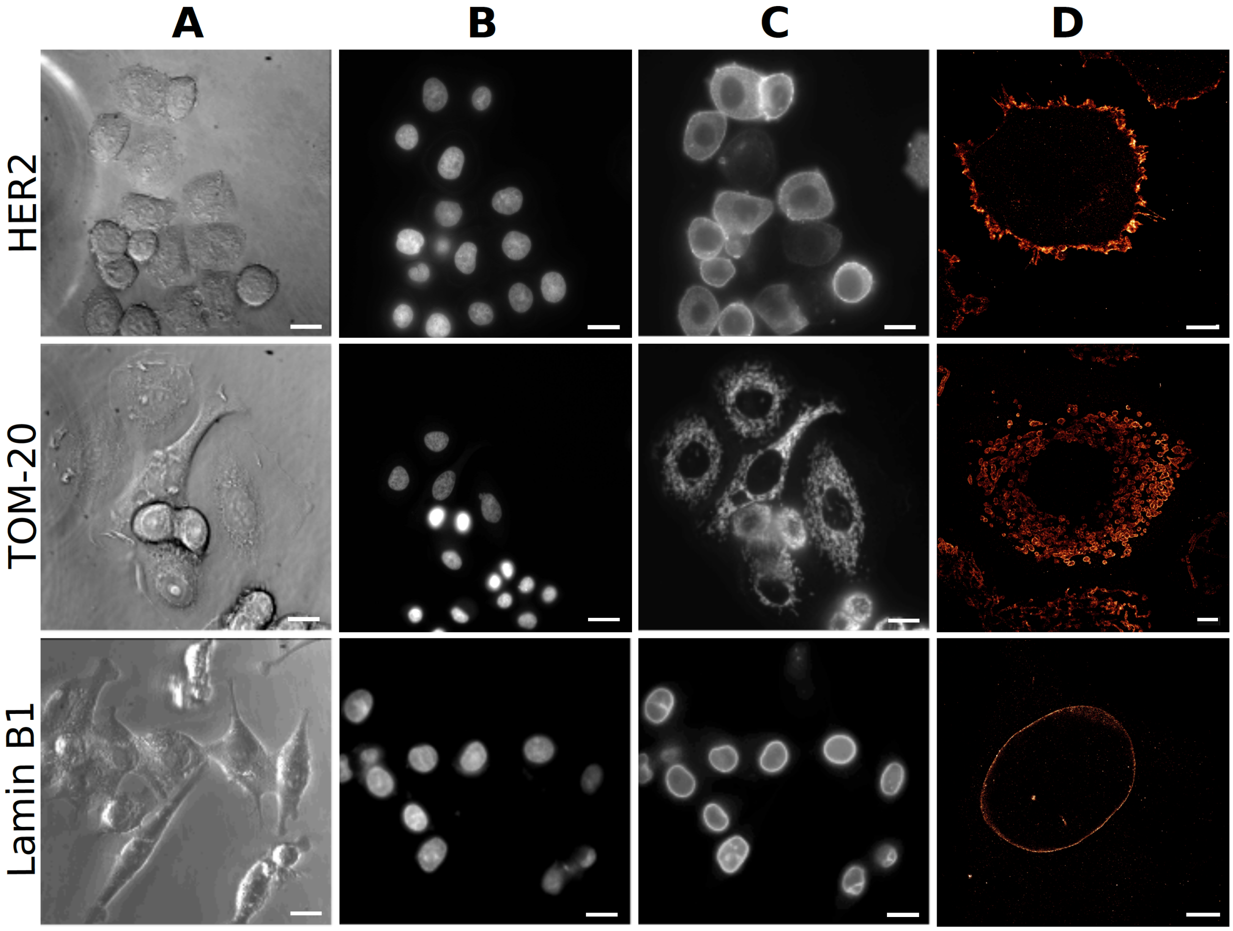

<u>Figure S1</u>: *Comparison of conventional and SMLM IF imaging in vitro*. Immunofluorescence staining of SKBR3 cells was completed using HER2, TOM-20, and Lamin B1. Images were collected at 40X magnification of **A.** bright field, **B.** DAPI, and **C.** IF using AF647 conjugated secondary antibody for detection. Scale bars = 10 µm. **D.** The same samples were imaged using SMLM to compare the ultrastructural features using each IF stain. Scale bars = 5 µm.

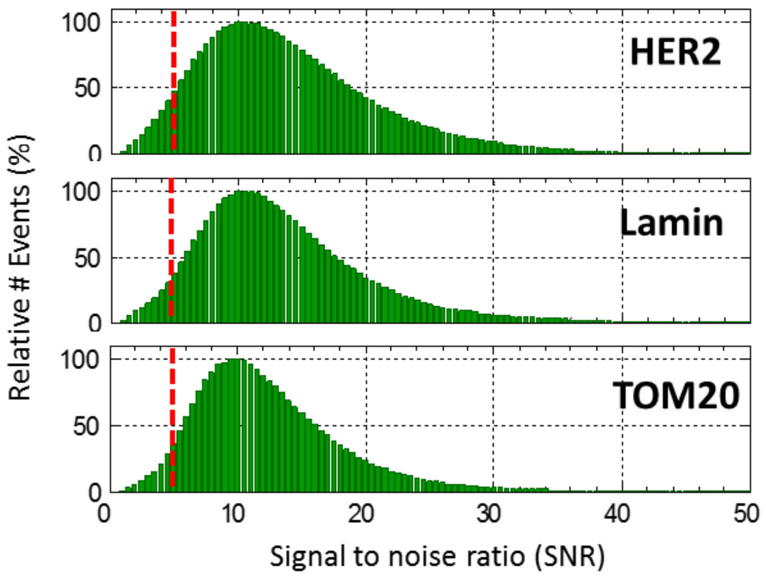

Figure S2. *Signal-to-noise ratio (SNR) analysis of the single-molecule images taken on 2 µm sections.* For each fluorescent molecule, a square (typically 9 x 9 pixels) region surrounding the molecule was used for Gaussian fitting. The signal for each single-molecule was calculated as the integrated area under the fitted Gaussian curve and above the background, and the noise was calculated as the square root of the residual sum of squares after Gaussian fitting. The histograms were derived from single-molecule images of AF647 on samples labeled for HER2 (top), Lamin (middle), and TOM20 (bottom) and normalized to the highest bin. The vertical dotted red line denotes the threshold of SNR used to filter out noisy localization events.

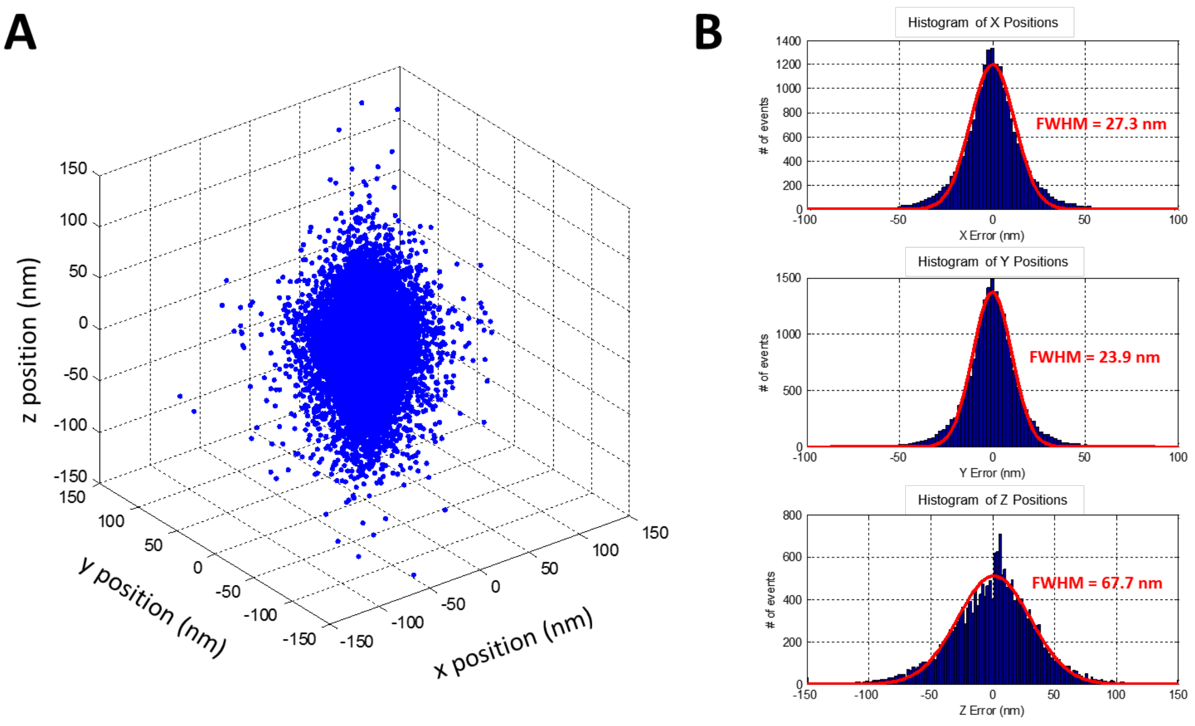

<u>Figure S3</u>. *Analysis of imaging resolution in all three dimensions.* Imaging resolution was calculated similarly to that reported by Huang *et al.*[1]. Briefly, each fluorescent molecule (AF647 in this case) generated a number of localization events in successive frames, each at a slightly different (x, y, and z) coordinate. An intensity-weighted average 3D position was calculated and used as the center-of-mass for this group of localization events, where the spread of individual coordinates around the center-of-mass reflected the localization precision of the molecule. (A) Coordinates of localization events from ~5,000 molecules (totaling 10,000-20,000 events) were aligned at the center-of-mass and plotted in 3D, generating a distribution of x, y, and z positions around (0, 0, 0). This particular dataset was from SMLM images of a 2 μm FFPE section immunostained for Lamin (data from HER2 and TOM20 were similar). (B) Histograms of x (top), y (middle), and z (bottom) positions shown in (A). The red curves are Gaussian fits to the histograms, with standard deviations of 11.9, 10.4, and 29.4 nm in x, y, and z, respectively. These

results correspond to FWHM (equivalent to spatial resolution at Nyquist limit) values of 27.3, 2.39, and 67.7 nm in the x, y, and z directions, respectively.

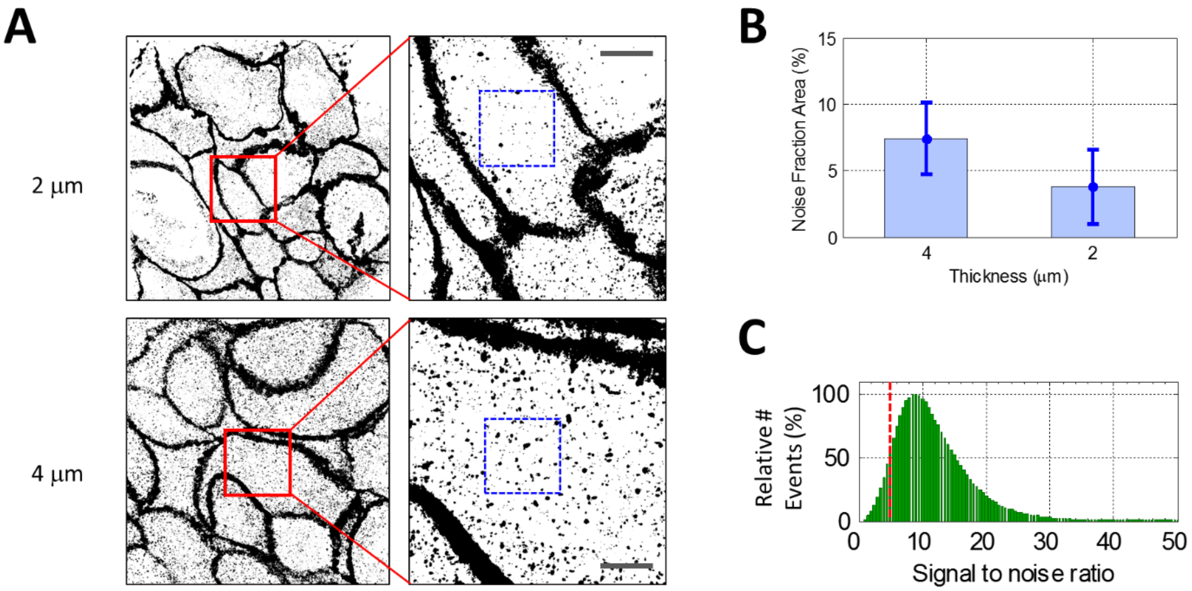

Figure S4. *Analysis of SMLM image background in 2 and 4 µm sections.* (A) SMLM images of HER2 (as shown in Figures 2-4) were first converted to binary images using Fiji[2] with automatic threshold values. The black regions indicated high intensity values (i.e., higher densities of localization events). The black outlines depict cell membranes where HER2 molecules were enriched, whereas events in the cytosol likely arose from background staining or imaging artifacts. Note that the effect was much more pronounced for 4 µm sections. Shown on the left are SMLM images of 35 × 35 μm^2 regions 2 (top) and 4 µm sections, and shown on the right are zoomed-in views of the boxed areas (red boxes, 10×10 μm^2). Blue, dashed boxes in the right panels indicate areas where signal was considered background; (B) Results of the fraction of area covered by background signal, analyzed as particles in the cytosol using the particle statistics function in Fiji. Distributions were obtained by analyzing multiple boxed regions, each approximately 3×3 mm^2 (A, right panel). Sample sizes for 4 and 2 µm sections are 17 and 13, respectively. Error bars indicate standard deviations; (C) SNR of single-molecule images of Alexa Fluor 647 taken on 4

μm sections labeled for HER2. Red dotted line denotes the SNR threshold used to filter out noisy localization events. Scale bars, 2 μm in (A, right panels).

REFERENCES

1. Huang, B., Wang, W., Bates, M. & Zhuang, X. Three-Dimensional Super-Resolution Imaging by Stochastic Optical Reconstruction Microscopy. *Science* **319**, 810-813 (2008).
2. Schindelin, J. et al. Fiji: an open-source platform for biological-image analysis. *Nat Methods* **9**, 676-682 (2012).
